# Predictors of reverse cardiac remodeling after sacubitril/valsartan in heart failure with reduced ejection fraction

**DOI:** 10.1038/s41598-026-36361-0

**Published:** 2026-01-30

**Authors:** Minjae Yoon, Soo Young Lee, Jin Joo Park, Sang-Eun Lee, Hyun-Jai Cho, Jin-Oh Choi, Byung-Su Yoo, Seok-Min Kang, Sue Lee, Dong-Ju Choi

**Affiliations:** 1 Division of Cardiology, Department of Internal medicine, Seoul National University Bundang Hospital, Seoul National University College of Medicine, Seongnam, Republic of Korea; 2 Division of Cardiology, Department of Internal Medicine, Asan Medical Center, University of Ulsan College of Medicine, Seoul, Republic of Korea; 3 Division of Cardiology, Department of Internal Medicine, Seoul National University Hospital, Seoul National University College of Medicine, Seoul, Seoul, Republic of Korea; 4 Department of Internal Medicine, Samsung Medical Center, Sungkyunkwan University College of Medicine, Seoul, Republic of Korea; 5 Department of Internal Medicine, Yonsei University Wonju College of Medicine, Wonju, Republic of Korea; 6 Division of Cardiology, Department of Internal Medicine, Severance Hospital, Yonsei University College of Medicine, Seoul, Republic of Korea; 7 Novartis Korea Limited, Seoul, Republic of Korea; 8 Cardiovascular Center, Internal Medicine, College of Medicine, Seoul National University Bundang Hospital, Seoul National University, 82, Gumi-ro 173 Beon-gil, Bundang-gu, Seongnam, 13620 Gyeonggi-do Republic of Korea

**Keywords:** Heart failure, Ventricular remodeling, Angiotensin receptor antagonists, Sacubitril/valsartan, Echocardiography, Cardiology, Medical research, Heart failure

## Abstract

Sacubitril/valsartan (Sac/Val) is associated with reverse cardiac remodeling in heart failure with reduced ejection fraction (HFrEF). However, the predictors of reverse cardiac remodeling after Sac/Val have not yet been fully established. We aimed to evaluate the predictors of reverse cardiac remodeling in patients with HFrEF, with a focus on HF duration and the dose of Sac/Val. In this retrospective, multicenter cohort study, 600 patients with HFrEF who received a Sac/Val prescription were enrolled at six tertiary hospitals in Korea between February 2017 and April 2019. After excluding patients without baseline or 12-month follow-up echocardiographic data, 294 patients were enrolled. Reverse cardiac remodeling was defined by comparing the baseline and follow-up echocardiographic data: an absolute increase in left ventricular ejection fraction (LVEF) ≥ 10% and a relative decrease in left ventricular end-diastolic volume index ≥ 10%. The average daily Sac/Val dose was calculated during the first 6 and 12 months after initiation. Among the 294 patients, 107 presented with reverse cardiac remodeling at 12 months. Patients with HF duration < 12 months at the time of Sac/Val initiation showed a higher proportion of reverse cardiac remodeling than patients with HF duration ≥ 12 months (46.1% vs. 25.7%; *P* < 0.001). Patients with an average daily Sac/Val dose ≥ 200 mg/day over 6 months also had a higher proportion of reverse cardiac remodeling than patients with Sac/Val < 200 mg/day (44.0% vs. 31.9%; *P* < 0.001). Multivariable logistic regression revealed low baseline LVEF, HF duration < 12 months, and higher Sac/Val dose as independent predictors of reverse cardiac remodeling. In conclusion, early initiation of Sac/Val following HF diagnosis and higher Sac/Val doses were associated with a higher likelihood of reverse cardiac remodeling in HFrEF.

**Supplementary Information:**

The online version contains supplementary material available at 10.1038/s41598-026-36361-0.

## Introduction

The PARADIGM-HF trial demonstrated the superiority of sacubitril/valsartan (Sac/Val) over enalapril in patients with heart failure (HF) with reduced ejection fraction (HFrEF)^1^. Subsequent studies including the PROVE-HF study showed that the use of Sac/Val in patients with HFrEF resulted in reverse cardiac remodeling, i.e., increased left ventricular (LV) ejection fraction (LVEF) and decreased LV chamber size^2–6^. Furthermore, a recent meta-analysis of more than 10,000 patients showed that Sac/Val is more effective at inducing reverse cardiac remodeling in HFrEF than angiotensin-converting enzyme inhibitor (ACEI) or angiotensin receptor blocker (ARB)^7^. Moreover, reverse cardiac remodeling after Sac/Val was associated with improved cardiovascular outcomes^6,8,9^, which has led to increased interest in reverse cardiac remodeling in HFrEF^10^.

However, only a few studies have investigated the predictors of reverse cardiac remodeling after Sac/Val, and they have been limited by small sample sizes or inconsistent results^3–5,11−13^. Predicting which patients are more likely to experience reverse cardiac remodeling with Sac/Val is important for treatment in real-world clinical practice. In addition, it remains unclear whether the timing of Sac/Val initiation and its dosage affect treatment efficacy and reverse cardiac remodeling.

Therefore, we aimed to evaluate the predictors of reverse cardiac remodeling in patients with HFrEF, focusing on HF duration and the Sac/Val dose.

## Methods

### Study design and population

This is a sub-study of the REal-world usAge of Sacubitril/valsartan in adult Korean heart failURE patients with reduced ejection fraction (REASSURE) study, which was published in October 2022^14^. The REASSURE study was a retrospective, multicenter cohort study that evaluated the real-world usage of Sac/Val in patients with HFrEF in Korea. Between February 2017 and April 2019, 600 patients with HFrEF who had received at least one Sac/Val prescription were identified by reviewing patient-level medical records from six tertiary hospitals in Korea.

Patients aged ≥ 18 years with HFrEF and at least 12 months of follow-up from the index date, defined as the date of the patient’s first Sac/Val prescription, were included in the study. HFrEF was defined as an LVEF ≤40% according to current guidelines^15–17^. Patients with less than 12 months of medical record activity were also included if they had died, received a heart transplant, or were in hospice or palliative care, with data up to the time of the event analyzed for reverse cardiac remodeling. All patients were treated with optimal medical therapy for HF, including renin-angiotensin system inhibitors, beta-blockers, and mineralocorticoid receptor antagonists, at the discretion of their treating physicians. An electronic case report form was used to document the data of the enrolled patients.

**Fig. 1:**
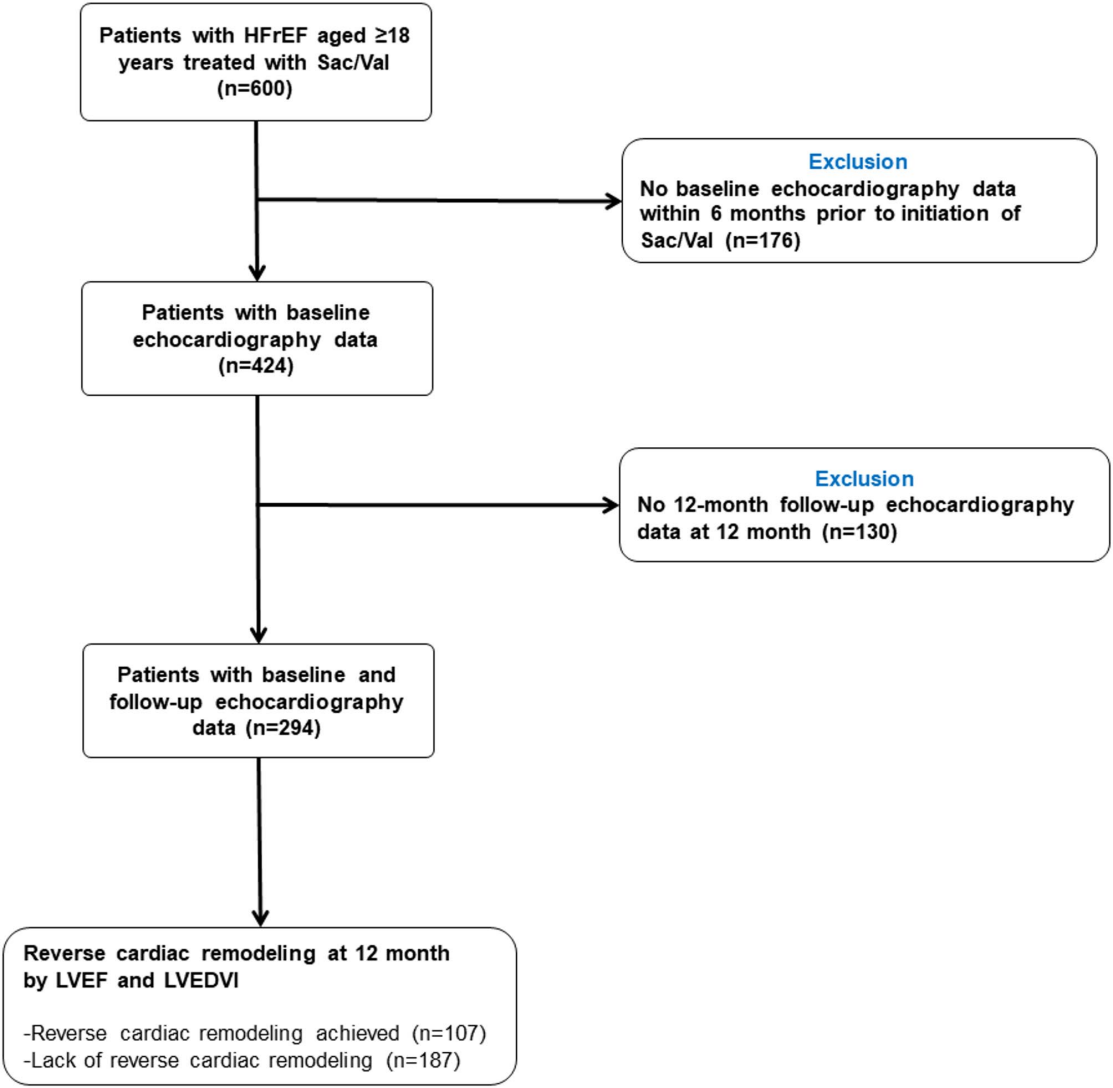
Study population flowchart. Among the 294 patients included in the analysis, 107 exhibited reverse cardiac remodeling and 187 did not. Reverse cardiac remodeling was defined as an absolute increase in LVEF ≥ 10% together with a relative decrease in LVEDVI ≥ 10% between baseline and follow-up echocardiographic assessments. HFrEF = heart failure with reduced ejection fraction; LVEDVI = left ventricular end-diastolic volume index; LVEF = left ventricular ejection fraction; Sac/Val = sacubitril/valsartan.

Patients were excluded from this study if they did not have baseline echocardiographic data within 6 months of starting Sac/Val therapy or if they missed a follow-up echocardiography at 12 months (Fig. 1). Echocardiographic re-evaluations not earlier than 6 months and not later than 18 months after the index date were used for the 12-month follow-up data. Finally, after excluding patients without baseline or follow-up echocardiographic data, 294 patients were included in this study.

The study was approved by the Institutional Review Board of Seoul National University Bundang Hospital (B2003/600 − 107) and the Institutional Review Boards of the other participating hospitals. All procedures were performed in accordance with relevant guidelines and regulations, including the Declaration of Helsinki. The requirement for informed consent was waived by the Institutional Review Board of Seoul National University Bundang Hospital (B2003/600 − 107) and the other Institutional Review Boards due to the retrospective nature of the study.

### Study variables

Information on demographic variables, medical history, physical examination, echocardiographic data, laboratory tests, and medications were collected. History of myocardial infarction (MI) was defined as a documented diagnosis in the medical records and confirmed by investigators at baseline, according to the universal definition of MI^18^. HF duration was defined as the difference between the date of the initiation of Sac/Val and the date of the first diagnosis of HF. Information on the Sac/Val dose was collected from baseline to 12 months. In addition, the average daily Sac/Val dose at 6 and 12 months after the index date was calculated using the following formula:

$$\:Average\:daily\:dosage\:of\:Sac/Val=\frac{Total\:dose\:prescribed\:after\:index\:date}{Total\:prescription\:days\:after\:index\:date}$$

Sac/Val 200 mg consists of 97 mg of sacubitril and 103 mg of valsartan. If a patient was taking Sac/Val 200 mg twice daily, the daily dose of Sac/Val was considered to be 400 mg/day. For patients who were not receiving Sac/Val at the end of follow-up, the last documented dose was used. Information regarding other HF medications was also collected during the follow-up period. Owing to the study’s retrospective design, HF medications, including Sac/Val, may have changed over time at the discretion of the treating physician.

### Echocardiographic data

All echocardiographic studies were performed using a standard ultrasound machine with a 2.5-MHz probe at each center. Standard images were obtained in the parasternal and apical views using two-dimensional, Doppler, and M-mode imaging. Echocardiographic parameters were measured according to the recommendations of the American Society of Echocardiography and the European Association of Cardiovascular Imaging^19^. LV end-diastolic diameter was calculated from M-mode or two-dimensional images. LV end-diastolic volumes (LVEDVs) and LV end-systolic volumes (LVESVs) were measured from apical four- and two-chamber views. The LV end-diastolic volume index (LVEDVI) and LV end-systolic volume index (LVESVI) were calculated as LVEDV and LVESV divided by body surface area. LVEF was measured using the modified Simpson’s biplane method and calculated as (LVEDV − LVESV) ×100 / LVEDV.

### Definition of reverse cardiac remodeling

Reverse cardiac remodeling, defined as an absolute increase in LVEF of at least 10% accompanied by a relative decrease in the LVEDVI of greater than 10%, was determined by comparing the baseline and 12-month follow-up echocardiographic data. We also performed a sensitivity analysis by adding a follow-up LVEF greater than 40% to the original definition of reverse cardiac remodeling. In another sensitivity analysis, the threshold for the relative decrease in LVEDVI was modified from ≥ 10% to ≥ 15%.

### Statistical analysis

Categorical variables are reported as frequencies (percentages) and were compared using Pearson’s chi-square test or Fisher’s exact test. Continuous variables were tested for normality using the Shapiro-Wilk normality test. Continuous variables with normal distribution are expressed as means ± standard deviations and were compared using Student’s *t*-test. Non-normally distributed continuous variables are presented as medians with interquartile ranges (IQRs) and were compared using the Mann–Whitney *U* test. Changes in echocardiographic parameters from baseline to 12 months were analyzed using paired *t*-tests. Logistic regression models were used to determine the predictors of reverse cardiac remodeling after Sac/Val. Multivariable logistic regression analyses were performed with the inclusion of clinically relevant variables according to previous studies or variables found to be statistically significant in the univariable analysis. Variables exhibiting multicollinearity with other included variables, determined as a variance inflation factor ≥4, were excluded from the analysis. HF duration and Sac/Val dose were assessed as either continuous or categorical variables, and the cutoff values for continuous variables were determined from the clinically relevant values.

All tests were two-tailed, and a *P*-value < 0.05 was considered statistically significant. Statistical analyses were performed using SPSS version 25.0 (IBM Corp., Armonk, NY, USA) and R programming version 4.2.0 (The R Foundation for Statistical Computing, Vienna, Austria).

## Results

### Baseline characteristics

The baseline characteristics of the study population are presented in Table 1. Among 294 patients, the mean age was 61.7 ± 13.5 years, 73.1% were male, 31.3% had hypertension, 30.6% had diabetes, and 22.4% had atrial fibrillation. The mean systolic and diastolic blood pressures were 115.7 and 68.8 mmHg, respectively. The median N-terminal pro-B-type natriuretic peptide (NT-proBNP) level was 1280 pg/mL [IQR, 445 to 3266 pg/mL], and the median baseline LVEF was 27.0% [IQR, 23.0% to 31.0%]. Regarding medications during follow-up, 86.1%, 58.2%, and 28.6% of patients received beta-blockers, mineralocorticoid receptor antagonists, and ivabradine, respectively.

**Table 1:** Patients’ baseline characteristics according to reverse cardiac remodeling at 12 months.

Variables	Total (*n* = 294)	Reverse cardiac remodeling (*n* = 107)	No reverse cardiac remodeling (*n* = 187)	*P* value
Demographics				
Age, years	61.7 ± 13.5	59.9 ± 13.6	62.8 ± 13.5	0.073
Male	215 (73.1%)	76 (71.0%)	139 (74.3%)	0.633
Weight, kg	66.4 ± 13.5	66.1 ± 15.5	66.6 ± 12.3	0.803
Height, cm	164.7 ± 9.0	165 ± 9.7	164.5 ± 8.6	0.637
Body mass index, kg/m^2^	24.3 ± 3.6	24.1 ± 3.9	24.5 ± 3.4	0.338
Comorbidities				
Hypertension	92 (31.3%)	31 (29.0%)	61 (32.6%)	0.604
Atrial fibrillation	66 (22.4%)	27 (25.2%)	39 (20.9%)	0.471
Dyslipidemia	49 (16.7%)	12 (11.2%)	37 (19.8%)	0.083
Diabetes	90 (30.6%)	30 (28.0%)	60 (32.1%)	0.553
Myocardial infarction	42 (14.3%)	8 (7.5%)	34 (18.2%)	0.019
HF duration, months	10.8 [2.4–52.8]	3.6 [1.2–25.2]	14.4 [3.6–60.6]	< 0.001
HF duration < 12 months	154 (52.4%)	71 (66.4%)	83 (44.4%)	< 0.001
NYHA classification				0.061
Class I	33 (11.2%)	8 (7.5%)	25 (13.4%)	
Class II	179 (60.9%)	70 (65.4%)	109 (58.3%)	
Class III	44 (15.0%)	20 (18.7%)	24 (12.8%)	
Class IV	5 (1.7%)	4 (3.7%)	1 (0.5%)	
Missing	33 (11.2%)	5 (4.7%)	28 (15.0%)	
Physical features				
Sitting SBP, mmHg	115.7 ± 17.5	117.2 ± 18.1	114.9 ± 17.1	0.267
Sitting DBP, mmHg	68.8 ± 13.3	70.4 ± 13.8	67.9 ± 13.0	0.128
Heart rate, beats/min	77.4 ± 14.1	78.6 ± 13.6	76.7 ± 14.3	0.263
Laboratory findings				
Hemoglobin, g/dL	13.7 ± 2.0	13.9 ± 2.2	13.5 ± 1.9	0.117
Blood urea nitrogen, mg/dL	18.1 [14.8–24.0]	18.0 [15.0–23.0]	18.2 [14.1–24.0]	0.677
Creatinine, mg/dL	1.0 [0.8–1.2]	1.0 [0.8–1.1]	1.0 [0.8–1.3]	0.174
eGFR, mL/min/1.73 m^2^	72.5 ± 26.2	75.6 ± 25.1	70.6 ± 26.7	0.127
eGFR < 60 mL/min/1.73 m^2^	81 (27.6%)	26 (24.8%)	55 (31.6%)	0.278
NT-proBNP, pg/mL (*n* = 118)	1280 [445–3266]	2155 [589–4921]	881 [432–2071]	0.031
Baseline echocardiography				
LVEF, %	27.0 [23.0–31.0]	23.7 [21.9–29.0]	28.4 [24.4–32.0]	< 0.001
LVEDD, mm	64 [59–69]	64 [59–69]	64 [60–69]	0.894
LVEDV, mL	188.5 [147.4–227.0]	199.0 [152.5–235.6]	183.7 [146.4–224.1]	0.224
LVEDVI, mL/m^2^	108.8 [86.3–132.5]	113.8 [89.4–137.7]	105.7 [83.8–128.4]	0.126
LVESV, mL	138.3 [104.0–175.0]	152.0 [109.5–182.5]	133.8 [101.0–164.5]	0.008
LVESVI, mL/m^2^	80.9 [60.8–99.4]	89.1 [66.9–106.3]	75.0 [58.3–93.9]	0.003
HF therapies during follow-up				
Beta-blockers	253 (86.1%)	94 (87.9%)	159 (85.0%)	0.619
MRA	171 (58.2%)	65 (60.7%)	106 (56.7%)	0.578
Ivabradine	84 (28.6%)	39 (36.4%)	45 (24.1%)	0.033
Sac/Val dose at baseline, mg/day	100 [100–200]	100 [100–200]	100 [100–200]	0.307
Average daily Sac/Val dose during 6 months, mg/day	153 [100–200]	184 [100–252]	139 [100–200]	0.017
Proportion of patients with an average daily Sac/Val dose ≥ 200 mg/day during 6 months	109 (37.1%)	48 (44.9%)	61 (32.6%)	0.049
Average daily Sac/Val dose during 12 months, mg/day	174 [100–239]	183 [100–290]	166 [100–210]	0.024
Proportion of patients with an average daily Sac/Val dose ≥ 200 mg/day during 12 months	109 (37.1%)	47 (43.9%)	62 (33.2%)	0.087

Values are expressed as mean ± standard deviations, median [25th–75th percentile], or n (%).

DBP = diastolic blood pressure; eGFR = estimated glomerular filtration rate; HF = heart failure; LVEDD = left-ventricular end-diastolic diameter; LVEDV = left ventricular end-diastolic volume; LVEDVI = left ventricular end-diastolic volume index; LVEF = left ventricular ejection fraction; LVESV = left ventricular end-systolic volume; LVESVI = left ventricular end-systolic volume index; MRA = mineralocorticoid receptor antagonist; NT-proBNP = N-terminal pro–B-type natriuretic peptide; NYHA = New York Heart Association; Sac/Val = sacubitril/valsartan; SBP = systolic blood pressure.

The changes in echocardiographic parameters from baseline to 12 months are presented in Table 2 and Fig. S1. Among the overall population, the median LVEF increased by 8.0% [IQR, 1.0% to 16.4%], from baseline values of 27.0% [IQR, 23.0% to 31.0%] to 12-month values of 35.2% [IQR, 29.0% to 44.6%]. Additionally, the median LVEDVI decreased by 15.8 mL/m^2^ [IQR, 1.6 to 33.1 mL/m^2^], from 108.8 mL/m^2^ [IQR, 86.3 to 132.5 mL/m^2^] to 87.3 mL/m^2^ [IQR, 66.4 to 109.2 mL/m^2^]. The median value of the relative change in the LVEDVI from baseline to 12 months was −13.4%.

**Table 2:** Change in echocardiographic parameters from baseline to 12 months.

	Baseline value	12-month value	Change from baseline to 12 months	*P* value (paired t-test)
Total population (*n* = 294)				
LVEF, %	27.0 [23.0–31.0]	35.2 [29.0–44.6]	8.0 [1.0–16.4]	< 0.001
LVEDD, mm	64 [59–69]	59 [54–65]	-4 [-8–0]	< 0.001
LVEDV, mL	188.5 [147.4–227.0]	154.5 [115.0–197.0]	-25.0 [-55.0 – -3.0]	< 0.001
LVEDVI, mL/m^2^	108.8 [86.3–132.5]	87.3 [66.4–109.2]	-15.8 [-33.1 – -1.6]	< 0.001
LVESV, mL	138.3 [104.0–175.0]	98.0 [63.0–133.0]	-28.6 [-65.0 – -8.3]	< 0.001
LVESVI, mL/m^2^	80.9 [60.8–99.4]	55.9 [36.5–75.8]	-17.7 [-37.2 – -4.9]	< 0.001
Reverse cardiac remodeling (*n* = 107)				
LVEF, %	23.7 [21.0–29.0]	46.0 [38.2–51.4]	19.0 [14.1–28.4]	< 0.001
LVEDD, mm	64 [59–69]	46 [38–51]	-8 [-13 – -5]	< 0.001
LVEDV, mL	199.0 [152.5–235.6]	118.5 [95.5–153.3]	-59.0 [-100.5 – -40.3]	< 0.001
LVEDVI, mL/m^2^	113.8 [89.4–137.7]	67.1 [57.1–87.8]	-34.6 [-56.9 – -22.7]	< 0.001
LVESV, mL	152.0 [109.5–182.5]	63.0 [45.5–93.2]	-79.0 [-109.5 – -50.0]	< 0.001
LVESVI, mL/m^2^	89.1 [66.9–106.3]	36.1 [28.2–51.1]	-44.8 [-60.7 – -30.2]	< 0.001
No reverse cardiac remodeling (*n* = 187)				
LVEF, %	28.4 [24.4–32.0]	31.0 [26.8–36.1]	2.5 [-0.5–7.0]	< 0.001
LVEDD, mm	64 [60–69]	62 [57–67]	-1 [-4–0]	< 0.001
LVEDV, mL	183.7 [146.4–224.1]	174.0 [135.0–213.4]	-9.6 [-28.0–5.0]	< 0.001
LVEDVI, mL/m^2^	105.7 [83.8–128.4]	98.1 [78.3–122.4]	-5.7 [-17.5–2.7]	< 0.001
LVESV, mL	133.8 [101.0–164.5]	119.2 [89.5–151.0]	-16.0 [-28.0–2.6]	< 0.001
LVESVI, mL/m^2^	75.0 [58.4–94.9]	67.2 [51.2–85.3]	-8.7 [-17.4–1.6]	< 0.001

Values are expressed as median [25th–75th percentile].

DBP = diastolic blood pressure; eGFR = estimated glomerular filtration rate; HF = heart failure; LVEDD = left-ventricular end-diastolic diameter; LVEDV = left ventricular end-diastolic volume; LVEDVI = left ventricular end-diastolic volume index; LVEF = left ventricular ejection fraction; LVESV = left ventricular end-systolic volume; LVESVI = left ventricular end-systolic volume index; MRA = mineralocorticoid receptor antagonist; NT-proBNP = N-terminal pro–B-type natriuretic peptide; NYHA = New York Heart Association; Sac/Val = sacubitril/valsartan; SBP = systolic blood pressure.

The distribution of Sac/Val doses and HF duration are presented in Table S1 and Fig. S2. At baseline, 7.5% started with less than 100 mg/day, 57.5% with 100–200 mg/day, 30.6% with 200–400 mg/day, and 4.4% with 400 mg/day of Sac/Val, respectively. Thirteen patients (4.4%) discontinued Sac/Val during follow-up and were not taking the medication at the end of the observation period. The average daily Sac/Val dose during the 6 months after initiation was 153 mg/day, and 37.1% received ≥ 200 mg/day of Sac/Val during the 6 months (Table 1). Similar results were shown regarding the dose over 12 months, with an average Sac/Val dose of 174 mg/day, and 37.1% of patients receiving ≥ 200 mg/day. The median HF duration was 10.8 months [IQR, 2.4 to 52.8 months], and 52.4% had an HF duration < 12 months.

### Reverse cardiac remodeling and echocardiographic parameters

Among the 294 patients, 107 (36.4%) presented with reverse cardiac remodeling at 12 months (Table 1). Patients with reverse cardiac remodeling were less likely to have MI (7.5% vs. 18.2%; *P* = 0.019). Prescription of ivabradine during follow-up was more prevalent in the reverse cardiac remodeling group (36.4% vs. 24.1%; *P* = 0.033). Patients with reverse cardiac remodeling had higher NT-proBNP levels (2155 pg/mL [IQR, 589 to 4921 pg/mL] vs. 881 pg/mL [IQR, 432 to 2071 pg/mL]; *P* = 0.031). In addition, patients with reverse cardiac remodeling were more likely to have a shorter HF duration than patients with no reverse cardiac remodeling (3.6 months [IQR, 1.2 to 25.2 months] vs. 14.4 months [IQR, 3.6 to 60.6 months]; *P* < 0.001). Moreover, both the average Sac/Val dose and the proportion of patients receiving ≥ 200 mg/day was higher in the reverse cardiac remodeling group.

The baseline LVEF was lower in the reverse cardiac remodeling group (23.7% [IQR, 21.9% to 29.0%] vs. 28.4% [IQR, 24.4% to 32.0%]; *P* < 0.001) (Table 2). The median absolute increase in LVEF from baseline to 12 months was higher in patients with reverse cardiac remodeling than in patients with no reverse cardiac remodeling (19.0% [IQR, 14.1% to 28.4%] vs. 2.5% [IQR, −0.5% to 7.0%]; *P* < 0.001). The reverse cardiac remodeling group showed a significantly greater relative decrease in LVEDVI during follow-up compared to the non-remodeling group (median relative change: −33.4% vs. −5.7%, *P* < 0.001; absolute change: −34.6 mL/m^2^ [IQR, −56.9 to −22.7 mL/m^2^] vs. −5.7 mL/m^2^ [IQR, −17.5 to −2.7 mL/m^2^]; *P* < 0.001).

### Reverse cardiac remodeling according to HF duration and average daily Sac/Val dose

**Fig. 2:**
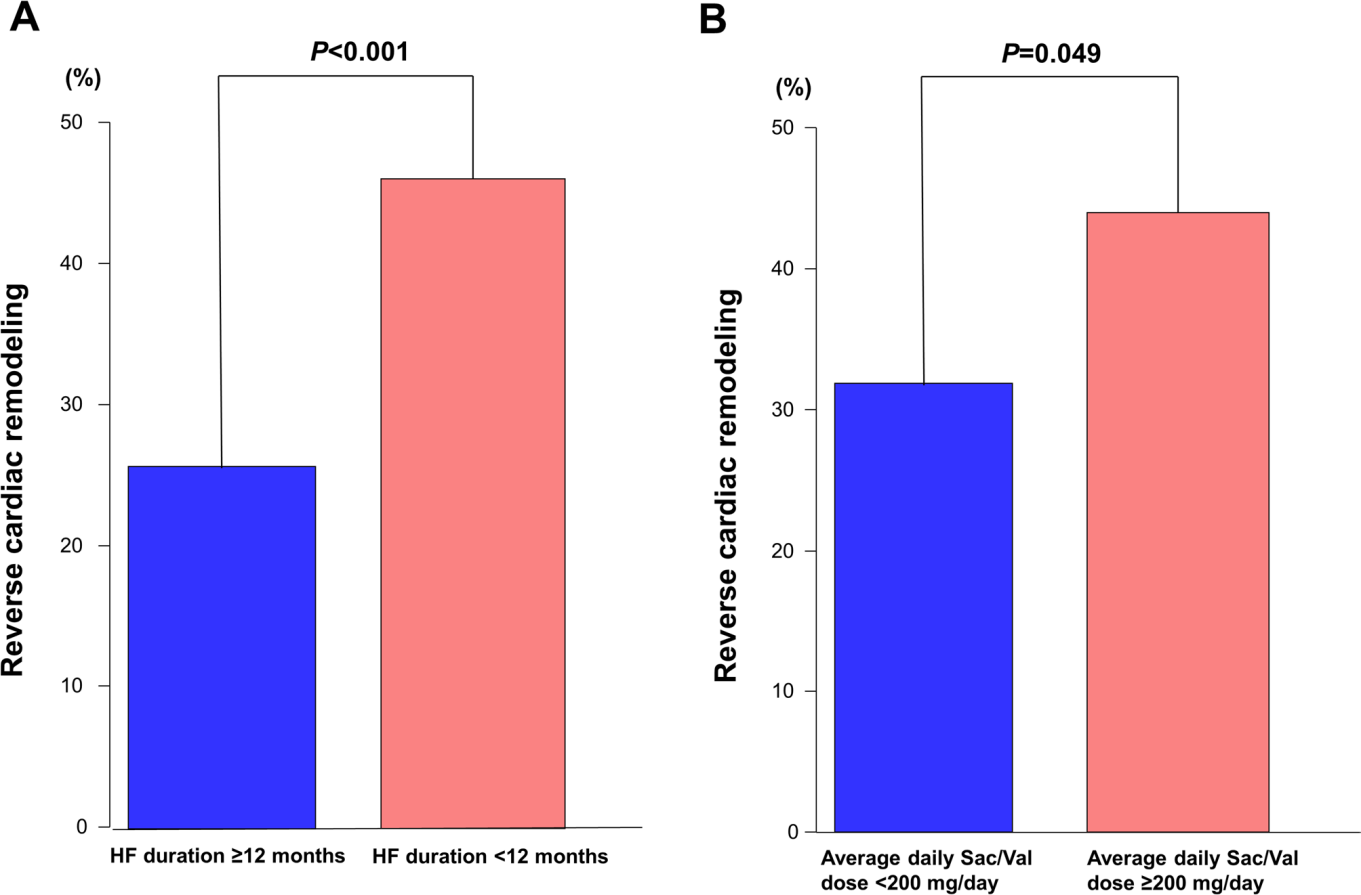
Reverse cardiac remodeling according to HF duration and average daily Sac/Val dose. (**A**) Patients with HF duration < 12 months at the time of Sac/Val initiation had a higher proportion of reverse cardiac remodeling than those with HF duration ≥ 12 months, (**B**) Patients with an average daily Sac/Val dose ≥ 200 mg/day over 6 months also showed a higher proportion of reverse cardiac remodeling than those with < 200 mg/day. HF = heart failure; Sac/Val = sacubitril/valsartan.

We used cutoff values of a 12-month duration of HF and an average Sac/Val dose of 200 mg/day to stratify patients into two groups. Patients with HF duration < 12 months at the time of initiation of Sac/Val showed a higher proportion of reverse cardiac remodeling at 12 months than patients with HF duration ≥ 12 months (46.1% vs. 25.7%; *P* < 0.001) (Fig. 2A). In addition, the absolute increase in LVEF from baseline to 12 months was higher in patients with HF duration < 12 months than in patients with no reverse cardiac remodeling (12.8% [IQR, 4.0% to 21.0%] vs. 5.0% [IQR, −0.2% to 12.0%]; *P* < 0.001). Patients with an average daily Sac/Val dose ≥ 200 mg/day had a higher proportion of reverse cardiac remodeling than patients with Sac/Val < 200 mg/day (44.0% vs. 31.9%; *P* < 0.001) (Fig. 2B).

Figure S3 presents the results of reverse cardiac remodeling according to HF duration and average daily Sac/Val dose, with patients stratified into three groups. When stratified by HF duration: ≥12 months, 3–12 months, and < 3 months, patients with a shorter HF duration were more likely to experience reverse cardiac remodeling (25.0% vs. 37.3% vs. 52.7%; P for trend < 0.001). When stratified by average daily Sac/Val dose: <100 mg/day, 100–199 mg/day, and ≥ 200 mg/day, there was an increasing trend in the achievement of reverse cardiac remodeling from patients on lower doses of Sac/Val to those on high doses of Sac/Val (29.5% vs. 32.6% vs. 44.0%; P for trend = 0.046).

### Predictors of reverse cardiac remodeling

The results of univariable logistic regression for the predictors of reverse cardiac remodeling after Sac/Val are presented in Table 3. In this model, MI, HF duration after diagnosis, NT-proBNP level, baseline LVEF, ivabradine use, and Sac/Val dose were significantly associated with reverse cardiac remodeling. History of MI was inversely associated with reverse cardiac remodeling, whereas higher NT-proBNP levels, lower baseline LVEF and ivabradine use were associated with increased likelihood of reverse cardiac remodeling. Notably, a shorter HF duration after diagnosis or an HF duration < 12 months was associated with a higher likelihood of reverse cardiac remodeling. In addition, a higher average daily Sac/Val dose during 6 or 12 months or an average daily dose ≥ 200 mg/day during 6 months was associated with a higher likelihood of reverse cardiac remodeling.

**Table 3:** Univariable logistic regression analysis for predictors of reverse cardiac remodeling.

Variables	Odds ratio	95% confidence interval	*P* value
Age, per 10 years	0.85	0.71–1.02	0.075
Age ≥ 65 years (vs. < 65 years)	0.66	0.41–1.07	0.096
Male (vs. female)	0.85	0.50–1.45	0.539
Body mass index, per kg/m^2^	0.97	0.90–1.03	0.337
Hypertension	0.84	0.50–1.41	0.517
Atrial fibrillation	1.28	0.73–2.24	0.387
Dyslipidemia	0.51	0.25–1.01	0.061
Diabetes	0.82	0.49–1.38	0.469
Myocardial infarction	0.36	0.15–0.78	0.014
HF duration, per year	0.92	0.86–0.98	0.013
HF duration < 12 months	2.47	1.52–4.08	< 0.001
NYHA classification ≥ III	1.65	0.88–3.09	0.117
SBP, per 10 mmHg	1.08	0.94–1.24	0.266
SBP ≥ 100 mmHg	1.03	0.56–1.95	0.926
SBP ≥ 110 mmHg	1.34	0.82–2.21	0.243
DBP, per 10 mmHg	1.15	0.96–1.38	0.130
Heart rate, per 10 beats/min	1.10	0.93–1.31	0.263
eGFR < 60 mL/min/1.73m^2^	0.71	0.41–1.22	0.223
NT-proBNP above median (*n* = 118)	2.39	1.13–5.18	0.025
LVEF, per 1%	0.88	0.83–0.92	< 0.001
LVEF < 25%	3.63	2.21–6.04	< 0.001
LVEF < 30%	2.20	1.30–3.80	0.004
LVEDD, per 10 mm	0.96	0.69–1.33	0.817
LVEDV, per 10 mL	1.01	0.98–1.05	0.558
LVEDVI, per 10 mL/m^2^	1.03	0.97–1.11	0.307
LVESV, per 10 mL	1.04	1.00–1.09	0.055
LVESVI, per 10 mL/m^2^	1.10	1.00–1.20	0.051
Beta-blocker	1.27	0.64–2.65	0.502
MRA	1.18	0.73–1.93	0.497
Ivabradine	1.81	1.08–3.04	0.025
Sac/Val dose at baseline, per 100 mg/day increment	1.15	0.84–1.58	0.371
Average daily Sac/Val dose during 6 months, per 100 mg/day increment	1.35	1.05–1.74	0.018
Average Sac/Val dose during 6 months ≥ 200 mg/day vs. <200 mg/day	1.68	1.03–2.74	0.037
Average daily Sac/Val dose during 12 months, per 100 mg/day increment	1.32	1.05–1.67	0.018
Average Sac/Val dose during 12 months ≥ 200 mg/day vs. <200 mg/day	1.58	0.97–2.58	0.066

DBP = diastolic blood pressure; eGFR = estimated glomerular filtration rate; HF = heart failure; LVEDD = left-ventricular end-diastolic diameter; LVEDV = left ventricular end-diastolic volume; LVEDVI = left ventricular end-diastolic volume index; LVEF = left ventricular ejection fraction; LVESV = left ventricular end-systolic volume; LVESVI = left ventricular end-systolic volume index; MRA = mineralocorticoid receptor antagonist; NT-proBNP = N-terminal pro–B-type natriuretic peptide; NYHA = New York Heart Association; Sac/Val = sacubitril/valsartan; SBP = systolic blood pressure.

The results of multivariable logistic analyses are summarized in Table 4. Low baseline LVEF (odds ratio [OR], 0.89; 95% confidence interval [CI], 0.84–0.93; *P* < 0.001), HF duration < 12 months (OR, 2.14; 95% CI, 1.24–3.74; *P* = 0.006), and higher average daily Sac/Val dose during 6 months (per 100 mg/day increment: OR, 1.39; 95% CI, 1.04–1.86; *P* = 0.028) were independent predictors of reverse cardiac remodeling after Sac/Val (Table 4, model 1). These results were consistent across variables related to the average Sac/Val dose used: (1) an average dose of ≥ 200 mg/day during 6 months (OR, 1.79; 95% CI, 1.03–3.12; *P* = 0.039; model 2), (2) average dose during 12 months per 100 mg/day increment (OR, 1.32; 95% CI, 1.01–1.73; *P* = 0.040; model 3), and (3) an average dose ≥ 200 mg/day during 12 months (OR, 1.64; 95% CI, 0.95–2.86; *P* = 0.075; model 4). Using HF duration as a continuous variable in the multivariable logistic models, shorter HF duration was the independent predictor of reverse cardiac remodeling, consistent with the main analyses (OR, 0.93; 95% CI, 0.86–0.99; *P* = 0.034) (Table S2).

**Table 4:** Multivariable logistic regression analysis for predictors of reverse cardiac remodeling.

Variables	Multivariable analysis 1			Multivariable analysis 2			Multivariable analysis 3			Multivariable analysis 4		
	OR	95% CI	*P* value	OR	95% CI	*P* value	OR	95% CI	*P* value	OR	95% CI	*P* value
Age, per 10 years	0.96	0.78–1.17	0.691	0.95	0.77–1.16	0.604	0.96	0.78–1.17	0.661	0.94	0.77–1.15	0.538
Male (vs. female)	0.83	0.45–1.54	0.561	0.83	0.45–1.54	0.558	0.84	0.46–1.55	0.572	0.86	0.47–1.58	0.621
Myocardial infarction	0.49	0.19–1.13	0.110	0.49	0.19–1.13	0.111	0.49	0.19–1.13	0.111	0.48	0.19–1.11	0.102
LVEF, per 1%	0.89	0.84–0.93	< 0.001	0.88	0.84–0.93	< 0.001	0.89	0.84–0.93	< 0.001	0.88	0.84–0.93	< 0.001
Ivabradine	1.59	0.87–2.88	0.127	1.56	0.86–2.81	0.140	1.61	0.89–2.92	0.117	1.60	0.88–2.88	0.120
HF duration < 12 months	2.14	1.24–3.74	0.006	2.10	1.22–3.65	0.008	2.14	1.23–3.72	0.007	2.09	1.22–3.63	0.008
Average daily Sac/Val dose during 6 months, per 100 mg/day increment	1.39	1.04–1.86	0.028									
Average Sac/Val dose during 6 months ≥ 200 mg/day vs. <200 mg/day				1.79	1.03–3.12	0.039						
Average daily Sac/Val dose during 12 months, per 100 mg/day increment							1.32	1.01–1.73	0.040			
Average Sac/Val dose during 12 months ≥ 200 mg/day vs. <200 mg/day										1.64	0.95–2.86	0.075

CI = confidence interval; OR = odds ratio; DBP = diastolic blood pressure; eGFR = estimated glomerular filtration rate; HF = heart failure; LVEDD = left-ventricular end-diastolic diameter; LVEDV = left ventricular end-diastolic volume; LVEDVI = left ventricular end-diastolic volume index; LVEF = left ventricular ejection fraction; LVESV = left ventricular end-systolic volume; LVESVI = left ventricular end-systolic volume index; MRA = mineralocorticoid receptor antagonist; NT-proBNP = N-terminal pro–B-type natriuretic peptide; NYHA = New York Heart Association; Sac/Val = sacubitril/valsartan; SBP = systolic blood pressure.

In a sensitivity analysis incorporating a follow-up LVEF greater than 40% into the definition of reverse cardiac remodeling, 73 of the 294 patients were classified as having reverse cardiac remodeling at 12 months. HF duration < 12 months (OR, 2.71; 95% CI, 1.49–5.07; *P* = 0.001) and higher average Sac/Val dose during 6 months (per 100 mg/day, OR, 1.32; 95% CI, 1.00–1.74; *P* = 0.049) were the independent predictors of reverse cardiac remodeling after Sac/Val in the multivariable logistic regression model, consistent with the main analyses. In another sensitivity analysis modifying the threshold for the relative decrease in LVEDVI from ≥ 10% to ≥ 15%, 96 of the 294 patients experienced reverse cardiac remodeling and, HF duration < 12 months (OR, 2.62; 95% CI, 1.49–4.69; *P* < 0.001) and higher average Sac/Val dose during 6 months (per 100 mg/day, OR, 1.40; 95% CI, 1.04–1.90; *P* = 0.027) remained the independent predictors of reverse cardiac remodeling, yielding similar results.

## Discussion

**Fig. 3:**
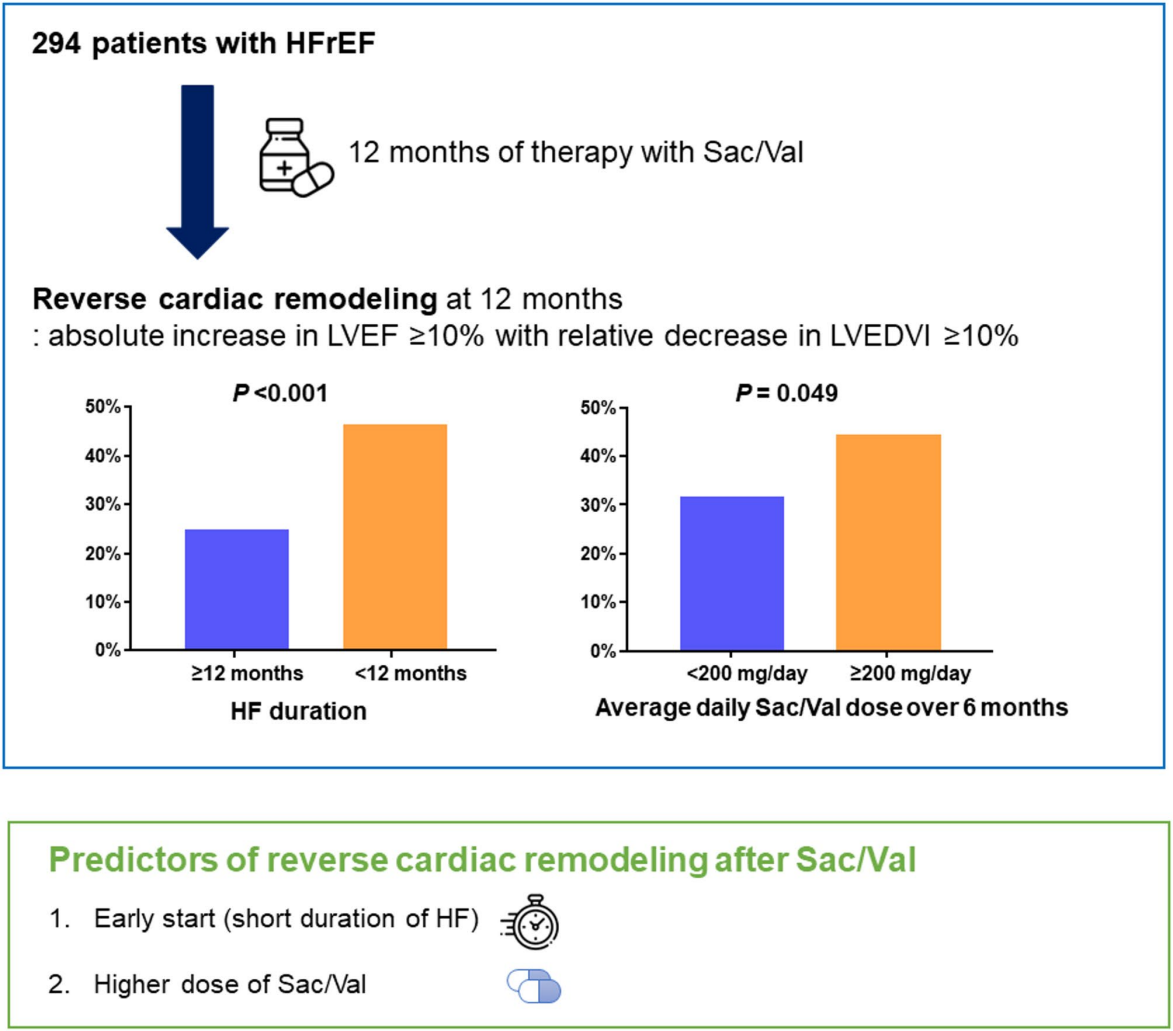
Key findings and predictors of reverse cardiac remodeling after Sac/Val. Shorter HF duration (< 12 months) and higher average Sac/Val dose (≥ 200 mg/day) were independent predictors of reverse cardiac remodeling in patients with HFrEF. HFrEF = heart failure with reduced ejection fraction; LVEDVI = left ventricular end-diastolic volume index; LVEF = left ventricular ejection fraction; Sac/Val = sacubitril/valsartan.

This multicenter retrospective cohort study evaluated the predictors of reverse cardiac remodeling in patients with HFrEF by focusing on HF duration and Sac/Val dose, and our principal findings were the following: (1) among patients with HFrEF with Sac/Val prescription, 36.4% showed reverse cardiac remodeling at 12 months; (2) patients with HF duration < 12 months or average daily Sac/Val dose ≥ 200 mg/day during 6 months had a higher chance of reverse cardiac remodeling; (3) low baseline LVEF, HF duration < 12 months, and higher Sac/Val dose were the independent predictors of reverse cardiac remodeling after Sac/Val in the multivariable logistic regression model (Fig. 3).

Reverse cardiac remodeling in HFrEF, a process where the LV geometry and/or function revert closer to those of a heart with normal structure and function, occurs in approximately one-third of cases, with rates ranging from 26% to 46%^6,20–22^, which is consistent with our findings. Traditionally, reverse cardiac remodeling in HFrEF has been associated with better clinical outcomes and prognosis^23^, and recent studies have also shown that reverse cardiac remodeling following Sac/Val is associated with improved cardiovascular outcomes^6,8,9^. As not all patients with HFrEF experience reverse cardiac remodeling, which is associated with a better clinical prognosis, it would be helpful to predict which patients are likely to have reverse cardiac remodeling. The identification of reliable predictors of reverse cardiac remodeling would help clinicians in adopting a more tailored approach, thus allowing closer follow-up and more rapid escalation of HF therapy for patients at higher risk of progressive LVEF decline and LV dilation^20^. Our results showed that patients receiving more aggressive HF medications, including early initiation or higher doses of Sac/Val, had a higher likelihood of reverse cardiac remodeling, suggesting that in addition to baseline characteristics such as LVEF or MI, treatment patterns during follow-up may also be associated with reverse cardiac remodeling. Moreover, prediction of reverse cardiac remodeling could help avoid premature and unnecessary implantation of an implantable cardioverter-defibrillator^24^.

After the introduction of Sac/Val, subsequent studies showed that the use of Sac/Val in patients with HFrEF also resulted in reverse cardiac remodeling^2–6^, and it was associated with marked improvements in LVEF and LV dimension compared with traditional ACEI or ARB therapy^7^. Sac/Val is a combination of an ARB and a neprilysin inhibitor that maintains neurohormonal balance and natriuretic peptide levels and may lead to better reverse cardiac remodeling than ACEI or ARB alone. Considering that Sac/Val is one of the four pillar medications in HFrEF^15,16,25^, predicting reverse cardiac remodeling after Sac/Val is clinically important; however, previous studies are scarce and have been limited by small sample sizes or inconsistent results^3–5,11−13^. Therefore, we performed an analysis of the predictors of reverse cardiac remodeling after Sac/Val using a multicenter cohort, especially focusing on HF duration and Sac/Val dose.

Previous studies on the predictors of reverse cardiac remodeling in HFrEF before the era of Sac/Val suggested that female sex, shorter HF duration, non-ischemic etiology, low baseline LVEF, and no late gadolinium enhancement on cardiac magnetic resonance imaging were important predictors^20^. Especially for HF duration, some recent small-scale retrospective studies after the era of Sac/Val have shown that shorter HF duration was associated with a higher likelihood of reverse cardiac remodeling after Sac/Val^3,5^. Another single-center retrospective study of 115 patients showed that earlier use of Sac/Val in de novo HF was associated with better clinical outcomes and earlier LV reverse remodeling^26^. A more recent post hoc analysis of the PROVE-HF trial showed that a longer HF duration was associated with less improvement in LVEF and LV chamber volume after the initiation of Sac/Val compared with a shorter HF duration^11^, consistent with our results. However, this post hoc analysis also indicated a small but significant improvement in cardiac size and function compared with baseline in patients with a long HF duration, supporting the apparent benefit of Sac/Val in these patients, given that most patients in the PROVE-HF trial had already been exposed to ACEIs or ARBs^2^. On the other hand, as the LIFE trial^27^ showed no benefit of Sac/Val in patients with advanced HF, further studies are warranted to clarify the efficacy of sacubitril/valsartan in long-standing or advanced stages of HF. Considering the results of previous studies and our own, the earliest possible initiation of Sac/Val in all patients with HFrEF, and especially in de novo HF, is clinically important to reverse cardiac remodeling.

There have been a few previous studies on Sac/Val dose and reverse cardiac remodeling. Martens et al.^4^ analyzed 125 patients with HFrEF in a single center and demonstrated a dose-dependent effect of Sac/Val on changes in LVEF and LVESV. However, another recent post hoc analysis of the PROVE-HF trial reported similar reverse cardiac remodeling according to the different Sac/Val doses during follow-up^12^. They reported that the median absolute LVEF improvement was 9.3%, 8.7%, and 10.2%, for low-, moderate-, and high-dose Sac/Val groups, respectively, slightly different from ours. We believe that this might be because the medication dose could be adjusted at the physician’s discretion, and the baseline characteristics and blood pressure differed according to medication dose. This discrepancy requires further analysis in a larger study. Interestingly, the Sac/Val dose achieved in clinical trials for HFrEF is often not achieved in real-world clinical practice^14^. For example, recent data have shown that only 14% of patients treated with usual care received the maximum Sac/Val dose (400 mg/day)^28^. Considering the results of previous studies and ours, Sac/Val may be associated with favorable reverse cardiac remodeling even at submaximal doses in patients with HFrEF; however, it is important to increase the Sac/Val dose to the maximum tolerated dose whenever possible^29^.

There is large heterogeneity in the definition of reverse cardiac remodeling^20,22,24^. This is because studies on reverse cardiac remodeling have used different imaging techniques, such as echocardiography or cardiac magnetic resonance imaging, and have considered changes in various measurements for defining reverse cardiac remodeling, including absolute LVEF increase, absolute LVEF cutoff, or relative change in LVESV or LVEDV, either alone or in combination^3,6,21,30,31^. In addition, the cutoff thresholds of these variables differ among studies; thus, currently, a standardized definition of reverse cardiac remodeling is lacking^20,22,24^. This variability in definitions, along with differences in patient characteristics, may explain the inconsistent predictors of reverse cardiac remodeling reported across studies. For example, in our study, patients with reverse cardiac remodeling had lower LVEF, higher baseline NT-proBNP levels, and comparable LV end-diastolic dimensions to those without remodeling, whereas other studies have reported different findings^24,32,33^. In light of previous studies and current consensus, we defined reverse cardiac remodeling using an absolute increase in LVEF and a relative decrease in the LVEDVI, reflecting both functional and volumetric improvement and aligning with the pathophysiological concept of reverse cardiac remodeling^2,3,21,24,31,34^. In addition, sensitivity analyses using modified definitions of reverse cardiac remodeling yielded results consistent with our main analysis, further supporting the robustness of our findings.

Recently, left atrial (LA) reverse remodeling has garnered increasing attention. While traditional studies on reverse cardiac remodeling have focused on ejection fraction and chamber size from the LV perspective, recent evidence^35^ suggests that LA dilation is associated with poor outcomes in HF, and that HF therapies may promote LA reverse remodeling. Moreover, a substantial proportion of patients with HFrEF exhibit LA reverse remodeling over a one-year period, which is strongly associated with improved prognosis. Although our study focused on the LV and did not analyze LA reverse remodeling in detail, future studies should explore reverse cardiac remodeling from the LA perspective as well as the LV.

### Limitations

This study has several limitations. First, this was a retrospective observational study, not a randomized trial; therefore, the results should be interpreted with caution, and the findings do not establish causality. In addition, this study included only East Asian patients, raising concerns about the generalizability of the results to other ethnic groups. Differences in genetic background, disease phenotype, and healthcare systems—including access, reimbursement, and practice patterns—may limit the generalizability of our findings. Moreover, as the study population consisted exclusively of patients treated with Sac/Val, the findings may not be generalizable to those in whom Sac/Val was not indicated, contraindicated, or not prescribed for other reasons. Second, although we used the average daily Sac/Val dose for 6 or 12 months in the analyses, the Sac/Val dose was not consistent and might have changed over time. In particular, blood pressure levels may have influenced dose adjustments, and patients in better condition may have been able to tolerate a higher doses of Sac/Val. Therefore, the observed association between higher Sac/Val dose and a greater likelihood of reverse cardiac remodeling should be interpreted with caution. Patients who tolerated and achieved higher doses may represent a less severely ill cohort, thereby introducing potential selection bias and residual confounding that cannot be completely ruled out. The requirement for 12-month follow-up echocardiography as part of the inclusion criteria may have preferentially selected more clinically stable and adherent patients, potentially influencing estimates of the incidence of reverse cardiac remodeling to some extent. Third, we were unable to collect detailed information on the ischemic etiology of HF, except for the presence of MI. In addition, the proportion of MI (14.3%) was relatively small; therefore, we could not accurately evaluate whether the ischemic etiology of HF affects reverse cardiac remodeling, albeit a history of MI was inversely associated with reverse cardiac remodeling in our univariable logistic regression model. Future studies with more detailed etiologic classification, including ischemic burden and myocardial viability, are warranted to better elucidate the interaction between HF etiology and reverse cardiac remodeling following sacubitril/valsartan. Fourth, HF medications may have changed during the follow-up period at the discretion of each physician, which could be a confounding factor. However, in our study, there were no significant differences in the use of beta-blockers and mineralocorticoid receptor antagonists during follow-up between patients with and without reverse cardiac remodeling. Despite these limitations, our study has the strength of being a multicenter cohort of patients with HFrEF on Sac/Val, with a particular focus on HF duration and Sac/Val dose. Therefore, our study provides important insights into predictors of reverse cardiac remodeling after Sac/Val.

## Conclusion

HF duration < 12 months and higher Sac/Val dose were independent predictors of reverse cardiac remodeling after Sac/Val in HFrEF. Thus, early initiation of Sac/Val following HF diagnosis and higher Sac/Val doses may play an important role in facilitating reverse cardiac remodeling in HFrEF.

## Supplementary Information

Below is the link to the electronic supplementary material.

Supplementary Material 1

## Data Availability

The data used to support the findings of this study are available from the corresponding author (Dong-Ju Choi) upon request.
