# Supplementary material for: Predictors of reverse cardiac remodeling after sacubitril/valsartan in heart failure with reduced ejection fraction
Source: Sci Rep. 2026 Jan 30;16:6731. doi: 10.1038/s41598-026-36361-0 (PMC12913927; doi:10.1038/s41598-026-36361-0)
Supplement: Supplementary file 1 — Supplementary Material 1 [file 41598_2026_36361_MOESM1_ESM.docx]

**Supplemental material**

**Table S1. Sac/Val dosing regimen at baseline and 12 month and average daily dose of Sac/Val**

| **Sac/Val dosing regimen** | **Baseline** | **12 months^a^** | **Average daily dose of**  **Sac/Val during 6 months** | **Average daily dose of**  **Sac/Val during 12 months** |
| --- | --- | --- | --- | --- |
| <100mg/day | 22 (7.5%) | 18 (6.1%) | 44 (15.0%) | 43 (14.6%) |
| 100mg/day to <200mg/day | 169 (57.5%) | 89 (30.3%) | 141 (48.0%) | 142 (48.3%) |
| 200mg/day to <400mg/day | 90 (30.6%) | 107 (36.4%) | 98 (33.3%) | 98 (33.3%) |
| 400mg/day | 13 (4.4%) | 78 (26.5%) | 11 (3.7%) | 11 (3.7%) |

Values are presented as n (%).

^a^For those who did not use Sac/Val at the end of follow-up, the last dose of Sac/Val was used.

Sac/Val=sacubitril/valsartan.

**Table S2. Multivariable logistic regression analysis for predictors of reverse cardiac remodeling using HF duration as a continuous variable**

| **Variables** | **Multivariable analysis 1** | | |  | **Multivariable analysis 2** | | |  | **Multivariable analysis 3** | | |  | **Multivariable analysis 4** | | |
| --- | --- | --- | --- | --- | --- | --- | --- | --- | --- | --- | --- | --- | --- | --- | --- |
|  | **OR** | **95% CI** | **P value** |  | **OR** | **95% CI** | **P value** |  | **OR** | **95% CI** | **P value** |  | **OR** | **95% CI** | **P value** |
| Age, per 10 years | 0.96 | 0.78–1.19 | 0.728 |  | 0.95 | 0.78–1.17 | 0.641 |  | 0.96 | 0.78–1.18 | 0.722 |  | 0.95 | 0.77–1.16 | 0.593 |
| Male | 0.81 | 0.44–1.49 | 0.495 |  | 0.81 | 0.44–1.48 | 0.489 |  | 0.81 | 0.45–1.49 | 0.503 |  | 0.83 | 0.46–1.52 | 0.546 |
| Myocardial infarction | 0.46 | 0.18–1.04 | 0.076 |  | 0.46 | 0.18–1.04 | 0.076 |  | 0.46 | 0.18–1.05 | 0.078 |  | 0.45 | 0.18–1.03 | 0.073 |
| LVEF, per 1 % | 0.88 | 0.84–0.92 | <0.001 |  | 0.88 | 0.83–0.92 | <0.001 |  | 0.88 | 0.84–0.93 | <0.001 |  | 0.88 | 0.84–0.92 | <0.001 |
| Ivabradine | 1.64 | 0.91–2.96 | 0.098 |  | 1.61 | 0.90–2.89 | 0.109 |  | 1.66 | 0.92–3.00 | 0.090 |  | 1.65 | 0.92–2.97 | 0.093 |
| HF duration, per year | 0.93 | 0.86–0.99 | 0.034 |  | 0.93 | 0.86–0.99 | 0.037 |  | 0.92 | 0.86–0.99 | 0.030 |  | 0.92 | 0.86–0.99 | 0.032 |
| Average daily Sac/Val dose during 6 months, per 100 mg/day increment | 1.35 | 1.04–1.85 | 0.028 |  |  |  |  |  |  |  |  |  |  |  |  |
| Average Sac/Val dose during 6 months ≥200 mg/day vs. <200 mg/day |  |  |  |  | 1.81 | 1.04–3.15 | 0.035 |  |  |  |  |  |  |  |  |
| Average daily Sac/Val dose during 12 months, per 100 mg/day increment |  |  |  |  |  |  |  |  | 1.34 | 1.00–3.25 | 0.034 |  |  |  |  |
| Average Sac/Val dose during 12 months ≥200 mg/day vs. <200 mg/day |  |  |  |  |  |  |  |  |  |  |  |  | 1.72 | 0.99–2.99 | 0.054 |

CI=confidence interval; HF=heart failure; LVEF=left ventricular ejection fraction; OR=odds ratio; Sac/Val=sacubitril/valsartan

**Figure S1. Distribution of the LVEF at baseline and follow-up**

**
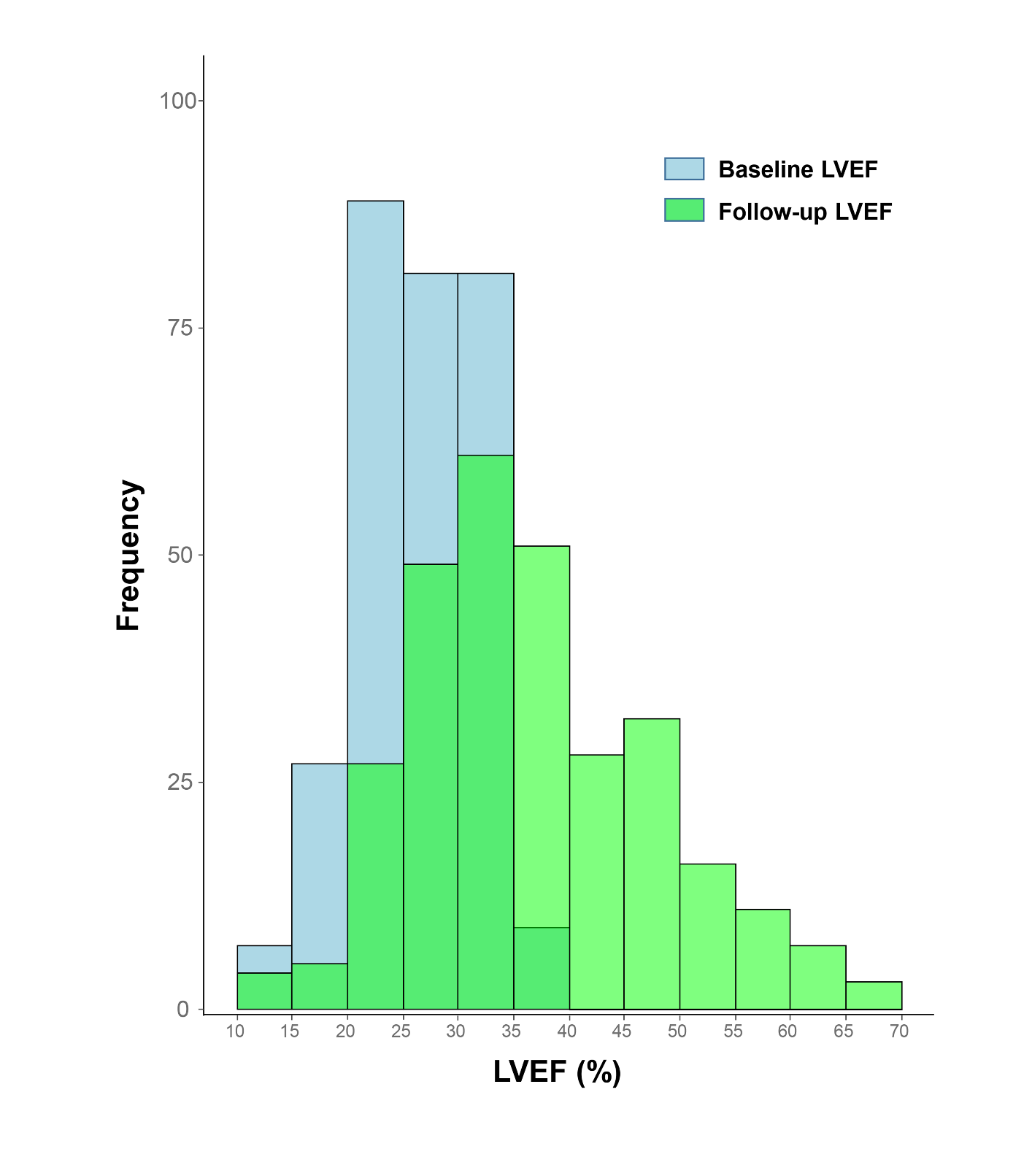
**

LVEF=left ventricular ejection fraction.

**Figure S2. Distribution of the HF duration and average daily Sac/Val dose**


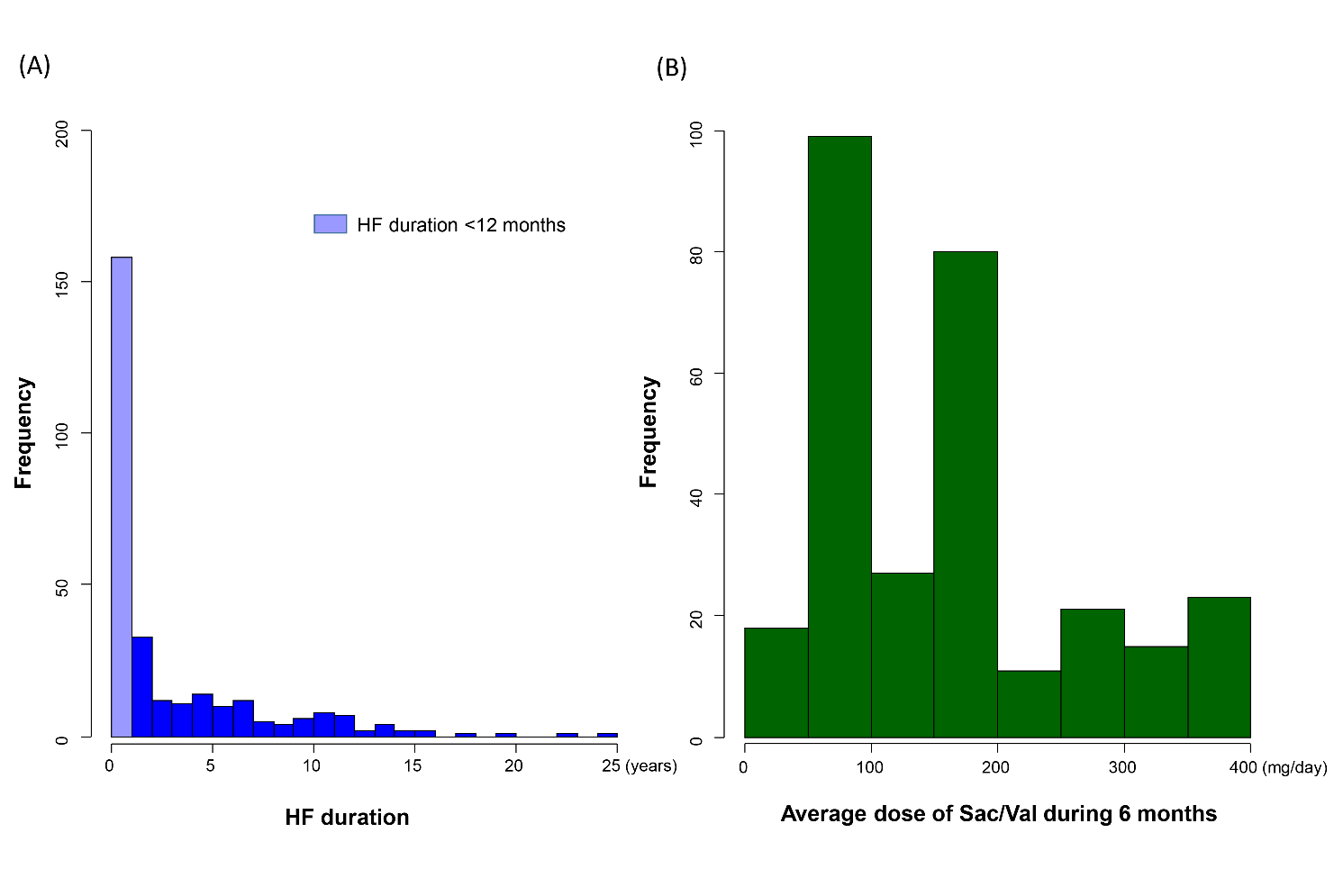


(A) HF duration, (B) average daily Sac/Val dose during 6 months.

HF=heart failure; Sac/Val=sacubitril/valsartan.

**Figure S3. Reverse cardiac remodeling according to HF duration and average daily Sac/Val dose stratified into three categories**


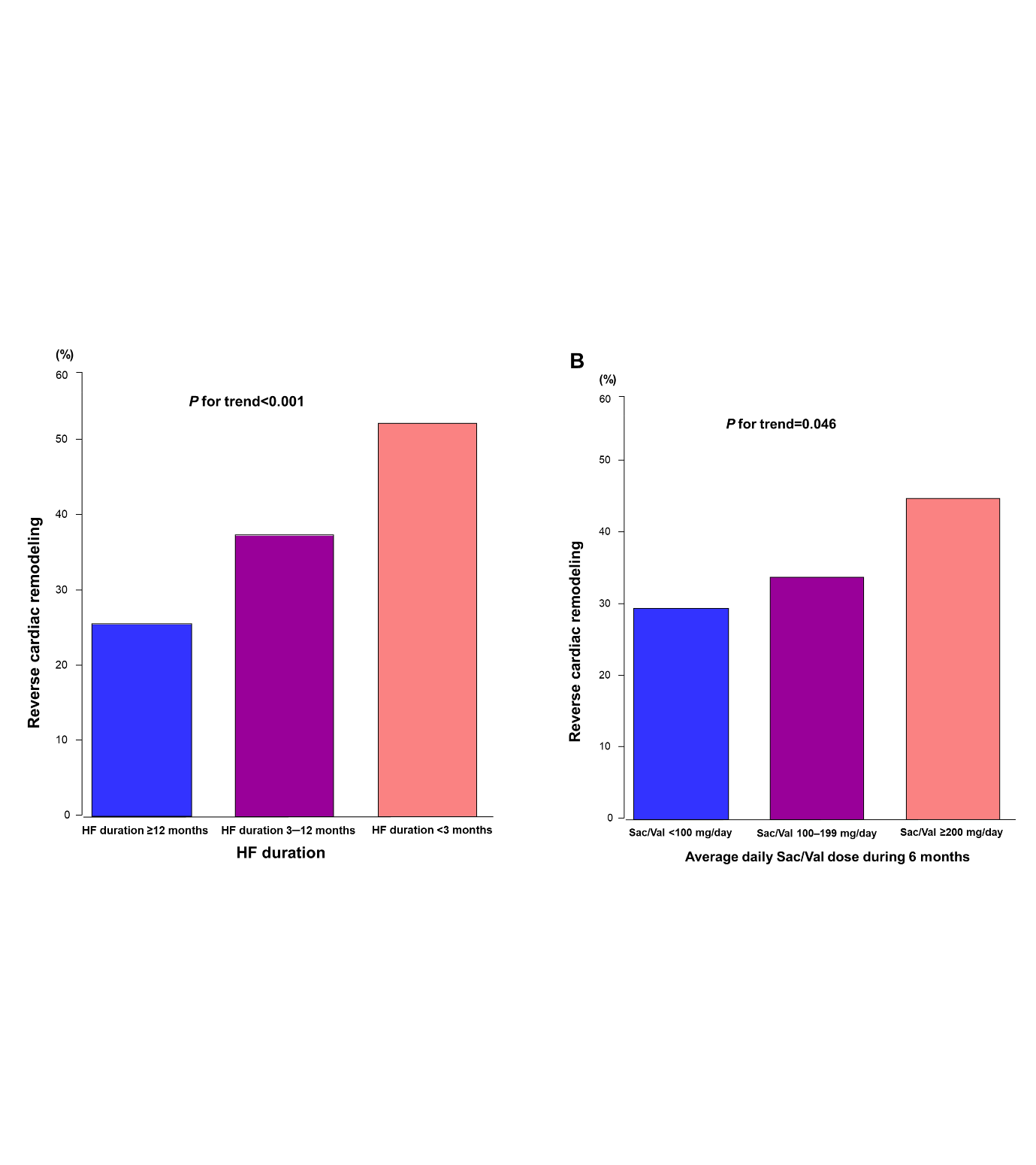


(A) According to HF duration and (B) average daily Sac/Val dose during 6 months.

HF=heart failure; Sac/Val=sacubitril/valsartan.
